# Synthesis and Evaluation of Tetramethylguanidinium-Polyethylenimine Polymers as Efficient Gene Delivery Vectors

**DOI:** 10.1155/2014/459736

**Published:** 2014-04-24

**Authors:** Manohar Mahato, Santosh Yadav, Pradeep Kumar, Ashwani Kumar Sharma

**Affiliations:** Nucleic Acids Research Laboratory, CSIR-Institute of Genomics and Integrative Biology, Delhi University Campus, Mall Road, Delhi 110 007, India

## Abstract

Previously, we demonstrated that 6-(N,N,N′,N′-tetramethylguanidinium chloride)-hexanoyl-polyethylenimine (THP) polymers exhibited significantly enhanced transfection efficiency and cell viability. Here, in the present study, we have synthesized a series of N,N,N′,N′-tetramethylguanidinium-polyethylenimine (TP1-TP5) polymers via a single-step reaction involving peripheral primary amines of bPEI and varying amounts of 2-(1H-benzotriazol-1-yl)-1,1,3,3-tetramethyluronium hexafluorophosphate (HBTU). These polymers were found to interact efficiently with negatively charged pDNA and formed stable complexes in the size range of ~240–450 nm. Acid-base titration profiles revealed improved buffering capacity of TP polymers as compared to bPEI. Transfection and cytotoxicity assays performed with TP/pDNA complexes on HEK293, CHO, and HeLa cells showed significantly higher transfection efficiency and cell viability with one of the complexes, TP2/pDNA complex, exhibited the highest transfection efficiency (~1.4–2.3-fold) outcompeting native bPEI and the commercially available transfection reagent, Lipofectamine 2000. Compared to previously reported THP polymers, the transfection efficiency of TP/pDNA complexes was found to be lower, as examined by flow cytometry. These results highlight the importance of the hydrophobic C-6 linker in THP polymers in forming compact nanostructures with pDNA, which might lead to efficient uptake and internalization of the complexes; however, the projected TP polymers offer an advantage of their rapid and economical one-step synthesis.

## 1. Introduction

Gene therapy has shown promising results in the treatment of both inherited and acquired genetic diseases; however, lack of a safe and efficient nucleic acid delivery system has hampered its widespread use [1–3]. Recently, nonviral vector-based nucleic acid delivery has gained great interest due to ease of its preparation, production, modification, low immunogenicity, and capability to deliver high molecular weight DNA molecules [4–8]. Among the variety of polymers, polyethylenimines (PEIs) are still considered as gold standard as these polymers exhibit high transfection efficiency. The prominent features of PEIs are intrinsic proton sponge property and high charge density due to the presence of 1°, 2°, and 3° amines (in ratio of 1 : 2 : 1), which enable these to condense DNA into small sized positively charged polyplexes that provide protection against enzymatic degradation and effective cell uptake. The secondary and tertiary amines provide buffering capacity for endolysosomal escape by facilitating endosomal disruption after endocytosis. High molecular weight bPEI (25 kDa) exhibits high transfection efficiency and is generally used as* in vitro* positive benchmark of gene carriers [9, 10]. However, high charge density (in particular, high density of primary amines) makes it interact nonspecifically with blood components leading to high cytotoxicity [11], which hinders its practical application in clinical gene therapy. To address these concerns, various modifications have been incorporated selectively on primary amines. Of these, hydrophobic modifications [12–18] have significantly improved cell viability and transfection efficiency by increasing pDNA condensation through cooperative binding as well as enhancing lipophilic interactions with cell membrane that promote pDNA release for transgene expression.

In our previous report [19], we tethered N,N,N′,N′-tetramethylguanidinium (TMG) groups on bPEI using a lipophilic C-6 linker in a multistep reaction. The incorporation of C-6 linker by blocking primary amines led to a decrease in zeta potential of the polyplexes although it was taken care of, to an extent, by the highly basic TMG groups. To overcome these concerns, we hypothesized that partial conversion of peripheral primary amines of bPEI into tetrasubstituted guanidinium moieties would result in the overall increase in the zeta potential and buffering capacity, which, in turn, would improve the transfection efficacy and cell viability (by reducing the density of 1° amines) of the modified polymers. Therefore, in order to preserve the charge density and incorporating lipophilicity in bPEI, we synthesized a series of N,N,N′,N′-tetramethylguanidinium-polyethylenimine (TP) polymers in a one-step reaction by treating bPEI with varying amounts of 2-(1H-benzotriazol-1-yl)-1,1,3,3-tetramethyluronium hexafluorophosphate (HBTU). Subsequently, these modified polymers were subjected to physicochemical characterization and found to condense pDNA into smaller sized particles, which were efficiently taken up by the cells and delivered the plasmid to its target without imparting cytotoxicity. The best performing formulation (TP2/pDNA) among these modified polymer complexes (in terms of transfection) was examined for its ability to protect bound pDNA against nucleases.

## 2. Experimental

### 2.1. Materials

Branched polyethylenimine (bPEI, 25 kDa), 3-(4,5-dimethylthiazol-2-yl)-2,5-diphenyltetrazolium bromide (MTT), 2-(1H-benzotriazol-1-yl)-1,1,3,3-tetramethyluronium hexafluorophosphate (HBTU), agarose, Tris, bromophenol blue (BPB), ethidium bromide (EtBr), and high retention dialysis tubing (12 kDa cut-off) were obtained from Sigma-Aldrich Chemical Co., USA. Bradford reagent was purchased from Bio-Rad Inc., USA. Lipofectamine 2000 was procured from Invitrogen Inc., USA. All other chemicals were of the highest purity and were procured locally. All the experiments were carried out using MilliQ (deionized) water, filtered through 0.22 *μ*m sterile filters (Millipore). For* in vitro* transfection assays, mammalian cell lines, namely, HeLa (human cervical adenocarcinoma), CHO (Chinese hamster ovary), and HEK293 (human embryonic kidney) cells, were maintained 16 h prior to the experiments as monolayer cultures in Dulbecco's modified Eagle's culture medium (DMEM) (GIBCO-BRL-Life Technologies, Web Scientific Ltd., UK) supplemented with 10% heat inactivated fetal bovine serum (FBS) (GIBCO-BRL-Life Technologies) and 50 mg/mL gentamycin. Cultures were maintained at 37°C in a humidified 5% CO_2_ atmosphere. The transfection experiments were carried out with the plasmid encoding enhanced green fluorescent protein (EGFP) gene (4.4 Kb) under the cytomegalovirus (CMV) immediate early promoter. The purity of the plasmid was confirmed by agarose gel electrophoresis and the ratio of UV absorbance at 260/280 in MilliQ water.

### 2.2. Instrumentation

All the new compounds were characterized by spectroscopic techniques. ^1^H-NMR was recorded in deuterated water on a Bruker Avance 400 MHz instrument and chemical shifts are reported in ppm. Size and zeta potential measurements were carried out on Zetasizer Nano-ZS (Malvern Instruments, UK). Green fluorescent protein (GFP) in transfected cells was assayed (*λ*_ex_: 488 nm; *λ*_em_: 509 nm) on NanoDrop ND-3300 spectrofluorometer (USA). The uptake and intracellular trafficking of dual labeled modified polymer/DNA complex were monitored by confocal microscopy (LSM 510 Meta Zeiss, Germany). Percent transfection efficiency was determined by flow cytometry (Guava EasyCyte Plus System, USA).

### 2.3. Preparation of N,N,N′,N′-Tetramethylguanidinium-Polyethylenimine (TP) Polymers 

In a solution of bPEI (30 mg, dissolved in 5 mL dry DMF) and TEA (6.07 *μ*L, 2.5 equivalents), HBTU (6.62 mg, 0.17 mmol for 10% substitution) was added and the reaction mixture was stirred for 12 h at ambient temperature. Then the reaction mixture was poured in a dialysis bag (12 kDa cut-off) and dialyzed against 1 M HCl solution for 24 h followed by against 1 M NaCl (pH 8.5) solution for 24 h and deionized water for 48 h with intermittent change of respective solutions/water. Subsequently, the dialyzed solution was lyophilized to obtain TP1 as white solid in ~78% yield. Similarly, other modified polymers, namely, TP2, TP3, TP4, and TP5, were prepared by varying the amounts of HBTU in 75–83% yield. These modified polymers were then characterized by ^1^H-NMR. TP5: ^1^H-NMR (D_2_O) *δ* (ppm): 2.4–2.65 (m, −NCH_2_−, PEI) 2.75 (br s, −NCH_3_, TMG).

### 2.4. Determination of Degree of Substitution in TP Polymers

Degree of substitution of tetramethylguanidinium moieties onto bPEI was determined by the standard 2,4,6-trinitrobenzenesulfonic acid (TNBS) assay [20]. Briefly, to an aqueous solution of a modified polymer (TP1, 100 *μ*L, 0.5 mg/mL) were added sodium hydrogen carbonate (1 mg) and a solution of TNBS (50 *μ*L, 0.1% in water). After incubating the reaction mixture at 40°C for 2 h, aqueous solutions of 10% sodium dodecyl sulphate (SDS, 50 *μ*L) and dilute HCl (1 N, 25 *μ*L) were added. Absorbance at 335 nm was recorded taking buffer of the same composition (without sample) as a blank. Degree of modification onto the polymer was calculated by drawing a standard curve using different concentrations of bPEI. Similarly, the degree of substitution of TMG groups onto other polymers (TP2–TP5) was determined.

### 2.5. Preparation and Physical Characterization of TP/pDNA Complexes

bPEI/pDNA and TP/pDNA complexes were prepared by separately mixing aqueous solutions of bPEI and TP polymers (1 mg/mL) with pDNA (1 *μ*L, 0.3 *μ*g/*μ*L) at their best working w/w ratio of 1.6 and 3.3, respectively, in water as well as in 10% FBS. The resulting complexes were vortexed and incubated for 30 min at ambient temperature prior to their use in physicochemical studies (size, morphology, and zeta potential).

Hydrodynamic diameter of bPEI/pDNA and TP/pDNA complexes, in water and 10% FBS, was recorded in triplicate by dynamic light scattering (DLS) using Zetasizer Nano-ZS. The data analysis was carried out in automatic mode and measured sizes were presented as the average values of 20 runs. Size and morphology of the TP2/pDNA complex (the best working formulation in terms of transfection efficiency) were also determined by high-resolution transmission electron microscopy (HR-TEM, Tecnai G2 30U-twin 300kV electron microscope). The sample, prepared at w/w ratio of 3.3 in water, was deposited on carbon-coated copper grids with 1% uranyl acetate negative staining and the image was captured at an accelerating voltage of 200 kV.

Similarly, zeta potential of bPEI/pDNA and TP/pDNA complexes, in water and 10% serum, was measured on Zetasizer Nano-ZS. Thirty runs were carried out in triplicate and the average values were estimated by Smoluchowski's approximation from electrophoretic mobility.

### 2.6. DNA Retardation Assay

Different amounts of TP polymers (1 mg/mL) were mixed with plasmid DNA (1 *μ*L, 0.3 *μ*g/*μ*L) to get final w/w ratio of 0.16, 0.33, 0.4, 0.5, 0.66, and 0.83 in 5% dextrose solution (5 *μ*L). Final volume of the samples was made up to 20 *μ*L with water, vortexed, and incubated for 30 min at ambient temperature. Then, these complexes were mixed with a tracking dye (xylene cyanol, 2 *μ*L), loaded on a 0.8% agarose gel and electrophoresed with Tris-acetate (TAE) buffer at 100 V for 45 min. Having stained the gel with ethidium bromide, it was visualized on a UV transilluminator using a Gel Documentation System (Syngene, UK) and imaged. Similarly, the assay was repeated with bPEI/pDNA complexes, prepared at w/w ratio of 0.16, 0.3, and 0.5 in 5% dextrose solution.

### 2.7. Buffering Capacity

Buffering capacity of modified polymers (TP1 to TP5) was determined by using the standard acid-base titration method [21]. The relative buffering capacity of polymers is usually estimated as amount of protons required to change pH from 10.0 to 3.0. Briefly, TP1 polymer (3 mg) was dissolved in 30 mL of 0.1 M NaCl and its pH was adjusted to pH 10.0 with 0.1 N NaOH and then the resulting solution was titrated with 0.1 N HCl by adding small aliquots (50 *μ*L) until pH 3.0 was reached. A graph was plotted between pH of the solution and the amount of 0.1 N HCl required to bring the pH of the solution from 10.0 to 3.0.

### 2.8. *In Vitro* Transfection Assay

In order to evaluate the gene carrying capacity of the TP polymers, transfection assay was carried out in the absence and presence of serum on various mammalian cell lines (HEK293, HeLa, and CHO cells) and the results were compared with those obtained using pDNA complexes of bPEI and the standard transfection reagent, Lipofectamine 2000. Briefly, HEK293 cells were seeded in 96-well plates at a density of 10,000 cells/well and cultured with medium containing 10% FBS for 16 h. Then the medium was aspirated, the cells were washed with phosphate buffer saline (1x PBS, 2 × 200 *μ*L), and the pDNA complexes of TP1 to TP5 were gently added, prepared at w/w ratio of 1.6, 2.6, 3.3, 5.0, and 6.6, bPEI at w/w ratio of 1.6, and Lipofectamine, prepared following the protocol provided by the manufacturer, onto the cells. Assay was carried out with pDNA complexes diluted with serum-free as well as serum containing DMEM. The plates were kept in an incubator at 37°C under humidified 5% CO_2_ atmosphere. After 3 h of incubation, transfection medium was replaced by fresh complete medium (100 *μ*L) and the cells were further incubated for 36 h to allow gene expression. Likewise, the assay was performed on HeLa and CHO cells. After 36 h of incubation, the plates were visualized under Nikon Eclipse TE 2000-S inverted microscope (Kanagawa, Japan) fitted with C-F1 epifluorescence filter (excitation, 488 nm; emission, 505 nm; barrier filter, BA 520) and the images of the cells expressed GFP were captured using Nikon Digital Imaging System. The transfection assay was carried out thrice to generate statistical data. Mock treated cells were used as blank.

### 2.9. Quantification of EGFP Expression

Estimation of expressed green fluorescent protein (GFP) was quantitatively carried out by measuring fluorescence at NanoDrop ND-3300 spectrofluorometer. Transfection was carried out as described above and after 36 h of incubation, cells were washed with 1x PBS (2 × 100 *μ*L) and cell lysis buffer was added (10 mM Tris, 1 mM EDTA, and 0.5% SDS, pH 7.4, 100 *μ*L). The plate was kept in an incubator for 30 min at 37°C and GFP content was determined in 2 *μ*L lysates spectrofluorometrically. The background and autofluorescence were corrected in mock treated cells. From each well, total protein content in the cell lysate was determined by Bradford's test taking bovine serum albumin as a standard. After subtraction of background, the fluorescence intensity was expressed in arbitrary units (a.u.)/mg of protein along with the mean ± standard deviation from triplicate samples.

### 2.10. Fluorescence-Activated Cell Sorting (FACS)

To find out transfection ability at individual cell level, HEK293 cells were seeded at approximately 20,000 cells/well in a 24-well plate and cultured for 16 h. TP1/pDNA, TP2/pDNA, TP3/pDNA (w/w ratio of 2.6, 3.3, and 5.0), THP2/pDNA (w/w ratio of 5), bPEI/pDNA (w/w ratio of 1.6), and Lipofectamine/pDNA (prepared following the manufacturer's protocol) complexes were prepared in DMEM and incubated at 37°C for 30 min. After aspiration of medium and washing of the cells with 1x PBS (2 × 1 mL), these complexes were gently added to respective wells and the plate was kept in an incubator at 37°C for 3 h. Then it was replaced by a fresh DMEM containing 10% FBS. After 36 h of incubation, medium was removed and cells were washed with 1x PBS (2 × 1 mL) followed by trypsinization with 300 *μ*L of trypsin-EDTA solution for 5 min. Cells were pelleted by centrifugation at 8000 rpm for 5.0 min at 4°C, washed with 1x PBS (2 × 1 mL), resuspended in 1x PBS, and transferred to flow cytometry cuvettes for analysis. The nontransfected cells were used as a negative control and adjusted such that no more than 1% cells fall above threshold fluorescence.

### 2.11. *In Vitro* Cytotoxicity Assay

*In vitro* cytotoxicity assay of the pDNA complexes of TP polymers, bPEI, THP2, and Lipofectamine was carried out on HEK293, HeLa, and CHO cells by MTT assay. Transfection assay was performed in serum-free conditions, as described above. After 36 h of incubation in a humidified 5% CO_2_ atmosphere at 37°C, the medium was aspirated and 100 *μ*L of MTT solution (1 mg/mL in DMEM) added to each well followed by incubation at 37°C for 3 h. Then the solution was removed, cells were washed with 1x PBS (2 × 100 *μ*L), and isopropanol (200 *μ*L) containing 0.04 M HCl and 0.5% SDS was added to each well. The plates were kept at ambient temperature for 30 min and the intensity of the color was measured spectrophotometrically at 540 nm after withdrawing aliquots from each well. Untreated cells were used as control having 100% viability and MTT reagent without cells was taken as blank to set autozero in the spectrophotometer. The relative metabolic activity of the cells (%) was calculated from the formula [absorbance]_sample_/[absorbance]_control_ × 100, where [absorbance]_sample_ denotes the optical density of the aliquot withdrawn from the well treated with the sample (polymer) and [absorbance]_control_ represents the optical density of the aliquot from the well treated with PBS only [22].

### 2.12. DNA Release Assay

To assess the DNA binding ability of TP2 polymer (the best system in the series in terms of transfection efficiency) and the stability of the resulting DNA complex, heparin release assay was carried out by gel electrophoresis and quantified the results densitometrically using Gene Tools Software from Syngene [23]. Briefly, TP2/pDNA and bPEI/pDNA complexes were prepared at their best w/w ratio of 3.3 and 1.6, respectively. After incubating these complexes for 30 min at ambient temperature, an aqueous solution of heparin (1 U/*μ*L) was added in increasing amounts (0–7.5 U) and the reaction mixture was incubated at ambient temperature for 30 min. The samples were then loaded on a 0.8% agarose gel, electrophoresed at 100 V for 1 h, and stained with ethidium bromide and the bands were visualized on a UV transilluminator using Gel Documentation System. The amount of DNA released from the complexes, after treatment with heparin, was calculated by densitometry.

### 2.13. DNase I Protection Assay

In order to examine the capability of the TP2 polymer to protect the condensed pDNA from nucleases present in the cellular milieu, DNase I protection assay was performed [24]. TP2/pDNA complex (prepared at w/w ratio of 3.3) and native pDNA were incubated at ambient temperature for 0.25, 0.5, 1, and 2 h with DNase I (1 *μ*L, 1 U/*μ*L in a buffer containing 100 mM Tris, 25 mM MgCl_2_, and 5 mM CaCl_2_). Similarly, these complexes were incubated with 1x PBS at ambient temperature, which served as controls. After completion of the exposure time, a solution of EDTA (1 *μ*L, 100 mM) was added to quench the activity of DNase I and degraded by heating at 75°C for 10 min. To release the bound pDNA from the polymer/pDNA complex, heparin (10 U, 1 U/*μ*L) was added and the complexes were incubated at ambient temperature for 30 min. Subsequently, samples were analyzed as described in the DNA release assay and the pDNA released from the complexes was estimated densitometrically.

### 2.14. Confocal Laser-Scanning Microscopy (CLSM)

To monitor the capability of TP2 to carry pDNA to the cytoplasmic and nuclear sites in the cells, confocal laser-scanning microscopy was used. Briefly, TP2 (4 mg), dissolved in double distilled (dd) water (1 mL), was allowed to react with 26 *μ*L of tetramethylrhodamine isothiocyanate (TRITC) (1 mg), dissolved in 100 *μ*L of DMF, for overnight to block ~1% of total amines. The solution was then subjected to concentration* in vacuo* and the residual unreacted/hydrolyzed TRITC was removed by trituration with ethylacetate (3 × 1 mL). Plasmid was labeled with YOYO-1 iodide. Briefly, plasmid DNA (1 *μ*L, 0.3 *μ*g/*μ*L) was mixed with YOYO-1 iodide (2 *μ*L, 1 mM solution in dimethylsulfoxide), stirred for 2 h at ambient temperature in dark, and stored at 4°C until used. HeLa cells were seeded at ~1.5 × 10^5^ cells/well on circular glass coverslips in a 6-well plate and incubated for 16 h. Tetramethylrhodamine- (TMR-) TP2/YOYO-1-pDNA complex (800 *μ*L) was added to each well and left for incubation for 0.25, 0.5, 1, and 2 h. Subsequently, the cells were subjected to washing with 1x PBS (3 × 0.5 mL) followed by fixing with 4% paraformaldehyde (PFA) for 10 min at 4°C. 4′,6-Diamidino-2-phenylindole (DAPI)), a blue nuclear dye, was used to counterstain the fixed cells and the coverslips were mounted over glass slides with fluorescence-free mounting medium (UltraCruz, Santa Cruz Biotechnology, USA). The images were captured using a Zeiss LSM 510 inverted laser-scanning confocal microscope.

## 3. Results and Discussion

**Scheme 1:**
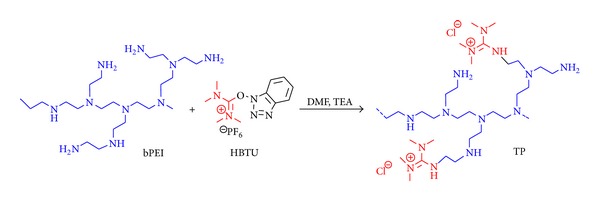
One-step synthesis of TP polymers.

As reported in our previous report, a multistep synthesis protocol yielded 6-(N,N,N′,N′-tetramethylguanidinium chloride)-hexanoyl-PEI (THP) polymers, which exhibited higher transfection efficiency and cell viability due to a decrease in the density of primary amines [19]. These modified polymers also showed tendency to self-assemble to form nanostructures. Further, to simplify the protocol, here, we directly reacted more accessible primary amines of bPEI with HBTU to obtain peripheral tetramethylguanidinium-PEI (TP) polymers. Varying the amounts of HBTU, a series of modified polymers (TP1–TP5) were synthesized (Scheme 1) in ~75–83% yield. Extent of substitution of tetramethylguanidinium groups on bPEI was determined by estimating the density of primary amines by the standard 2,4,6-trinitrobenzene sulphonic acid (TNBS) assay (Table 1) [20], which was found to be ~15–39% of the attempted substitution. As tetramethylguanidinium (TMG) moiety has a hydrophobic shell of four methyl groups, the percent substitution was found to decrease on increasing the concentration of HBTU due to steric hindrances.

### 3.1. Physicochemical Studies of TP/pDNA Complexes

**Figure 1:**
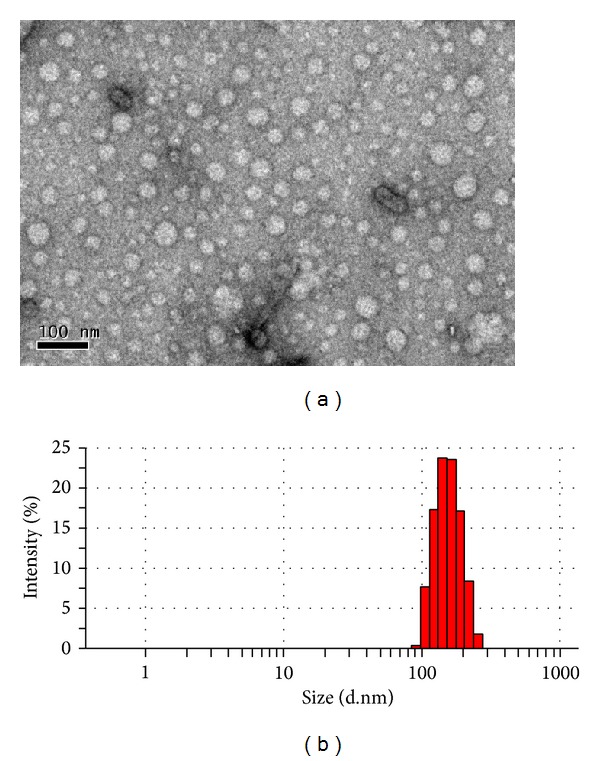
(a) TEM image and (b) size measurement (by DLS) of TP2/pDNA complex at w/w ratio of 3.3.

Modified polymers were examined for their ability to form polyplexes with the help of DLS. Size and zeta potential of TP/pDNA complexes were measured at their best w/w ratio of 3.3 in water and 10% FBS. The size of the complexes was found in the range of ~240–450 nm (*ca.* 124–155 nm in case of THP/pDNA complexes), while, in 10% FBS, it decreased to ~50–130 nm (Table 2). This decrease in size might be attributed to the change of medium from water to 10% FBS, which led to restricted hydration of nanoparticles and reduced tendency of aggregation as serum proteins stabilized individual complex particle and inhibited particle-particle interaction [21, 25–27]. The morphology and size of TP2/pDNA complex were also examined by transmission electron microscopy (TEM) at w/w of 3.3; particles were found to be spherical in shape with average size of ~60 nm (Figure 1) (*ca. *~26.6 nm in case of THP2/pDNA complex). The difference in size measured by DLS and TEM was due to measurement of hydrodynamic diameter in DLS, while TEM measures size of the particles in dry state [14, 19].

It was envisaged that incorporation of tetramethylguanidinium moiety (pKa 15.2) [19, 28, 29] would increase the zeta potential of polymers; however, down the series (TP1 to TP5), it decreased. pDNA complexes of TP1 (+43.2 mV) and TP2 (+38.3 mV) showed higher zeta potential than bPEI/pDNA complex (+27.8 mV), while TP3–TP5 complexes displayed lower zeta potential than bPEI complexes. This trend could be explained on the basis of shielding of primary amines by bulky tetramethylguanidinium groups, which rendered them inaccessible on the surface for measurements. These results were fully supported by electrophoretic mobility shift assay where TP1, TP2 retarded pDNA at lower concentration as compared to bPEI, while TP3–TP5 required higher amounts of polymers to retard the same amount of pDNA. Further, all the TPs complexes showed negative zeta potential in the presence of serum as reported in the literature [30].

### 3.2. Gel Retardation Assay for TP Polymers

**Figure 2:**
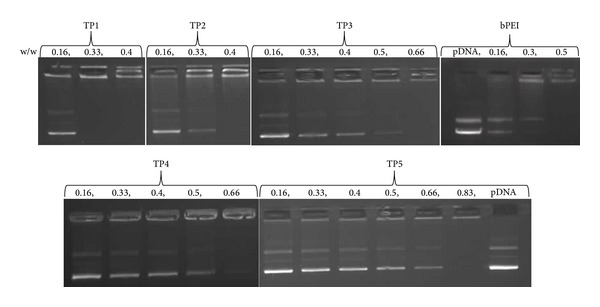
Gel retardation assay for TP/pDNA and bPEI/pDNA complexes at different w/w ratios.

To demonstrate the ability of the modified polymers to bind pDNA, gel retardation assay was done. TP/pDNA complexes were prepared at various w/w ratios and mobility of pDNA was observed on an agarose gel.  Figure 2 depicts pDNA migration under the influence of electric current. bPEI retarded the complexed pDNA at w/w ratio of 0.5, while the same amount of pDNA was retarded by TP polymers at different w/w ratio; that is, TP1 and TP2 retarded at 0.33 and 0.4, and both TP3, TP4 retarded at 0.66, while TP5 retarded at w/w ratio of 0.83. These results were in accordance with DLS study as TP1, TP2 showed higher zeta potential, which allowed them to retard pDNA mobility at lower amounts than bPEI, while TP3, TP4, and TP5 retarded the same amount of pDNA at higher amounts. This behaviour of polymers might be due to partial shielding of primary amines by bulky tetramethylguanidinium groups, which affected the accessibility of functional groups to interact with pDNA [19, 25, 28].

### 3.3. Tetramethylguanidinium-Polyethylenimine Polymers Exhibited Higher Buffering Capacity

**Figure 3:**
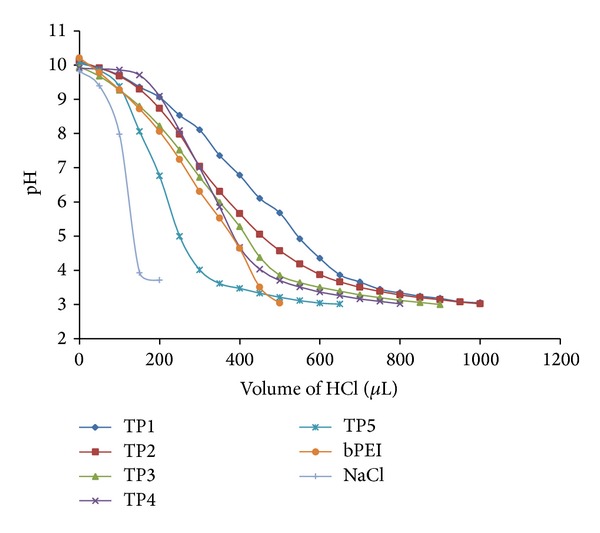
Buffering capacity of bPEI and TP polymers was determined by an acid-base titration method.

Buffering capacity of polymers was considered as an important parameter in the class of nonviral gene delivery vectors, which determines the release of vector-pDNA in the cytoplasm after endocytosis of complex. After conjugation of tetramethylguanidinium groups, buffering capacity of TP polymers increased as compared to bPEI, due to high pKa value (~15.2) of tetramethylguanidinium moiety [28, 29]. It is quite evident from Figure 3 that TP polymers required higher volume of 0.1 N HCl to bring pH from 10.0 to 3.0 compared to bPEI, but, within TP polymers, a decrease in buffering capacity was observed as modification of amine groups increased from TP1 to TP5. This might be due to the steric hindrance caused by bulky hydrophobic TMG moieties, which might have prevented accessibility for protonation of the TP polymers [19, 28].

### 3.4. TP/pDNA Complexes Showed Enhanced Transfection Efficiency

**Figure 4:**
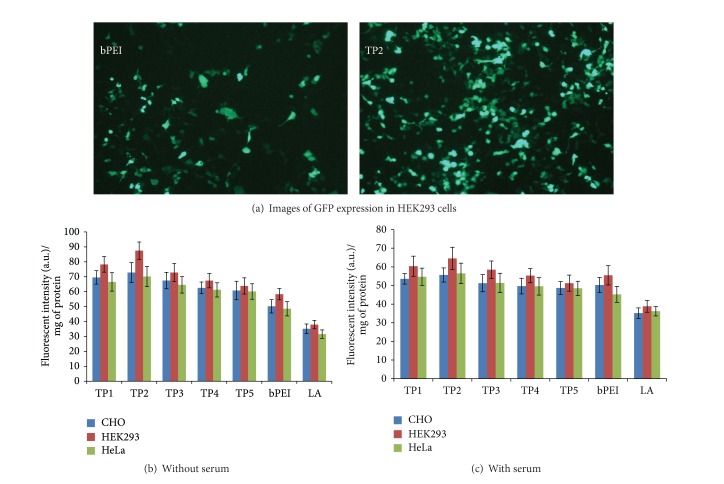
*In vitro* transfection assay. (a) Images of GFP expression after transfection with bPEI/pDNA and TP2/pDNA complexes in HEK293 cell at w/w ratio of 1.6 and 3.3, respectively. CHO, HEK293, and HeLa cells were transfected with pDNA complexes of TP polymers, bPEI, and Lipofectamine (LA) in the absence (b) and presence (c) of serum. Cells were incubated with complexes for 3 h and the expression was monitored after 36 h. The results represent the mean of three independent experiments performed in triplicate.

The capability of TP polymers to carry pDNA inside the cells was evaluated on HEK293, CHO, and HeLa cells in the absence and presence of serum by quantitative estimation of expressed GFP using NanoDrop spectrofluorometer. Complexes of TP polymers and bPEI were prepared at different w/w ratios, while Lipofectamine/pDNA complex was prepared following the manufacturer's protocol. All the complexes were incubated for 30 min and gently added on the cells. After 36 h of incubation, all the cells showed fairly high level of GFP gene expression and Figure 4(a) shows images of GFP expression in HEK293 cells. Initially transfection efficiency in both serum-free and serum containing assays increased with increasing w/w ratio from 1.6 to 3.3 and after attaining the maximum at w/w ratio of 3.3, it decreased. Among all the TP polymers complexes, TP2/pDNA formulation, at w/w ratio of 3.3, exhibited the highest transfection efficiency in all three cell lines (Figure 4(b)). In HEK293 cells, TP2/pDNA complex exhibited ~1.4–2.3-fold (in absence of serum, Figure 4(b)) and ~1.1–1.7-fold (in presence of serum, Figure 4(c)) higher transfection efficiency than bPEI and Lipofectamine complexes. In presence of serum, all the formulations showed significant decrease in transfection efficiency due to interaction of serum components with the modified polymers (Figure 4(c)). Still, among all the formulations, TP2/pDNA complex displayed the highest transfection efficiency in all three cell lines. The decrease in transfection efficiency subsequent to hydrophobic modifications was also reported by various research groups using cationic polymers as gene delivery vectors (15, 17, 18, and 31). Higher transfection efficiency of TP/pDNA complexes might be due to contribution from various factors, such as higher surface charge (zeta potential) and buffering capacity, which might have facilitated the release of complexes from the endosomes to cytoplasm for nuclear translocation as well as higher cell viability.

### 3.5. TP/pDNA Complexes Exhibited Higher Transfection

**Figure 5:**
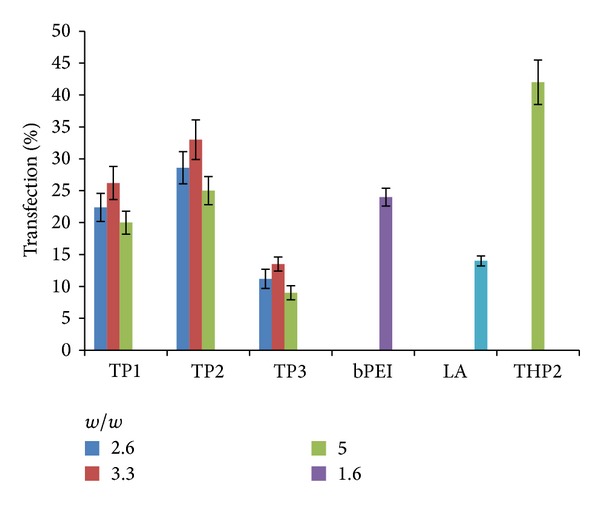
Percent transfection efficiency of pDNA complexes of TP1, TP2, TP3, THP2, bPEI, and Lipofectamine (LA) on HEK293 cells.

Quantitative estimation of transfection in cells was carried out on HEK293 cells by flow cytometry.  Figure 5 depicts the percent transfection of TP1, TP2, and TP3 complexes at w/w ratio of 2.6, 3.3, and 5.0, as well as pDNA complexes of THP2 at w/w ratio of 5.0, bPEI at 1.6, and Lipofectamine as per the manufacturer's protocol. Nontransfected cells were used as negative control. Among all TP/pDNA complexes, TP2 exhibited the highest percent transfection in cells at w/w ratio of 3.3, that is, ~33%. bPEI and Lipofectamine/pDNA complexes showed ~24% and 14% transfected cells, respectively. In our previous study [19], THP2/pDNA complex with C-6 linker showed ~42% transfected HEK293 cells, which might be attributed to smaller size of the complex that could be uptaken and internalized better as compared to TP/pDNA complexes. Other factors could be easy unpackaging of the THP2/pDNA complex inside the cells resulting in higher gene expression and influence of lipophilic C-6 linker allowing THP functional groups more accessibility for interaction with the cell membranes facilitating the entry of the complex inside the cells.

### 3.6. TP/pDNA Complexes Displayed High Cell Viability

**Figure 6:**
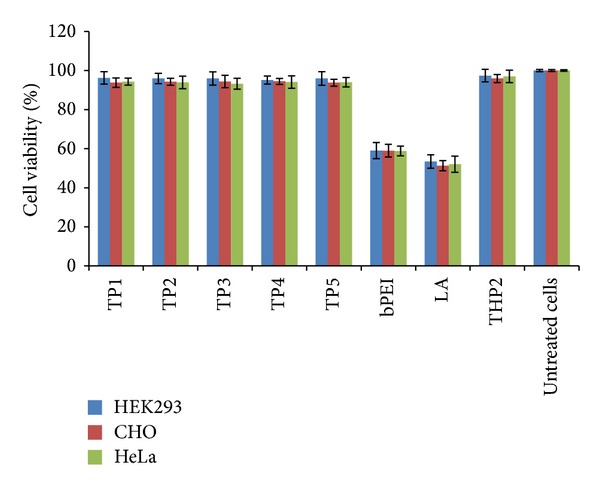
Cell viability assay of pDNA complexes of bPEI, THP2, TP1–TP5, and Lipofectamine (LA) on HEK293, CHO, and HeLa cells.

Cationic polymers induce cytotoxicity mainly due to excessive charge density imparted by primary amines. They aggregate on cell surface with the help of strong electrostatic binding, which usually destabilize and ultimately impair the function of cell membrane.  Figure 6 shows the results of MTT assay on HEK293, CHO, and HeLa cells for pDNA complexes of TP polymers, bPEI, and Lipofectamine. All the TP/pDNA formulations irrespective of percent substitution exhibited significantly higher cell viability than bPEI and Lipofectamine complexes in all the three cell lines as in case of THP complexes [19]. The increase in cell viability of the projected TP polymers might be explained on the basis of partial conversion of primary amines to tetramethylguanidinium (delocalized cationic charge). Moreover, the TMG group induces hydrophobicity (hinders nonspecific interactions with the cell membrane) and steric hindrance to the primary amines, which make them less accessible to interact with cell membrane. Thus reducing the availability of primary amines by converting them into substituted guanidinium moieties considerably lowered the toxicity of pDNA complexes.

### 3.7. TP2 Polymer Efficiently Releases pDNA

**Figure 7:**
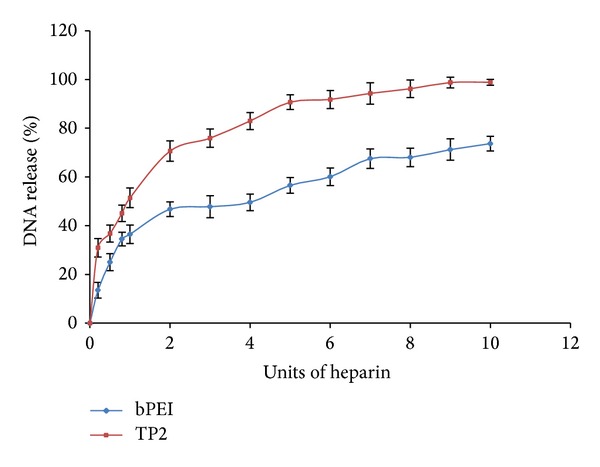
pDNA release assay of bPEI and TP2 polymers. Heparin was added in increasing amounts to pDNA complexes of bPEI and TP2 and the released pDNA was run on a 0.8% agarose gel and quantified.

The unpackaging of nucleic acids from vector/pDNA complex plays an important role in transfection efficiency. In principle, a cationic vector should bind pDNA sufficiently enough to provide protection against nucleases and loose enough to release it in cytoplasm for nuclear translocation [31]. In the present investigation, stability of TP2/pDNA complex (prepared at w/w ratio of 3.3) was evaluated by DNA release assay using heparin on an agarose gel and quantitative analysis of DNA bands was carried out densitometrically [23].  Figure 7 shows that, on increasing heparin units, pDNA release also increased. Addition of 8.0 U of heparin resulted in ~90% release of pDNA, while, with the same amount of heparin, bPEI could release ~60% of bound pDNA. These results clearly suggested the capability of TP2 polymer to unpackage the nucleic acid efficiently by disassembly of the complex in comparison with bPEI, leading to higher transfection efficiency by these modified polymers.

### 3.8. TP2 Provides Protection to pDNA against Enzymatic Degradation

**Figure 8:**
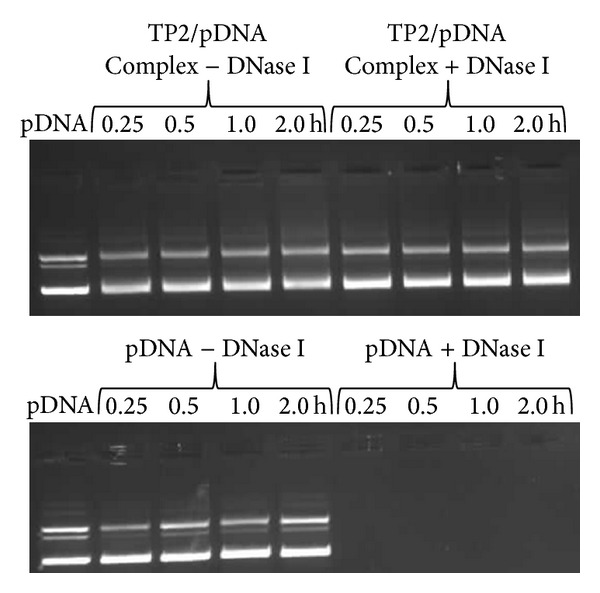
DNase I protection assay. TP2/pDNA complex, prepared at its best working w/w ratio of 3.3, was treated with 1 U of DNase I for 0.25, 0.5, 1.0, and 2.0 h. Complexes were treated with 20 U of heparin to release the pDNA from complex, which was run on a 0.8% agarose gel at 100 V for 45 min and stained with ethidium bromide. Released DNA was quantified densitometrically.

To evaluate the ability of TP2 polymer to provide protection to the bound pDNA against nucleases present in the cellular milieu [32], DNase I assay was performed. The results (Figure 8) showed that naked pDNA got completely degraded within 15 min by 1 U of DNase I, while the pDNA condensed with TP2 at w/w ratio of 3.3 was stable against enzyme even after exposure for 2 h (~90% of the pDNA was intact, as measured by densitometry). It is inferred that TP polymers are also capable of providing protection to bound pDNA against DNase I enzyme, which is considered to be one of the important requisites for efficient gene delivery.

### 3.9. Intracellular Trafficking of TP2/pDNA Complex

**Figure 9:**
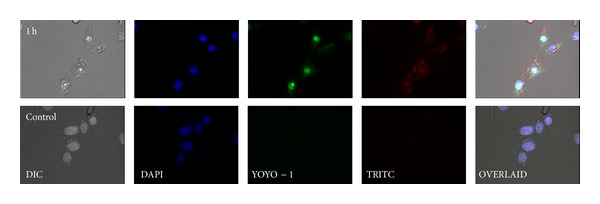
CLSM images of HeLa cells treated with a TMR-TP2/YOYO-1-pDNA complex for 1.0 h. Nucleus was stained with DAPI and the cells were observed under different filters.

To monitor the internalization and intracellular localization of the dual labeled TP2/pDNA complex (the best system in terms of transfection efficiency) in HeLa cells, confocal microscopy was used (Figure 9). TP2 was labeled with TMR and pDNA with YOYO-1. The complex (TMR-TP2/YOYO-1-pDNA) was prepared at the best w/w ratio of 3.3 and added onto the cells. After 1 h of exposure, a significantly large number of particles entered the nucleus, which was stained with DAPI exhibiting blue fluorescence to distinguish from green and red fluorescence shown by pDNA and vector system, respectively, indicating the potential of TP2 to carry pDNA to the nucleus of the cell (Figure 9). This could be explained on the basis of charge-mediated and hydrophobic interactions between tetramethylguanidinium groups TP and lipidic constituents of the cell membranes, which facilitated the internalization of the complex to the interiors of the cells.

## 4. Conclusions

In the present study, we have prepared a series of N,N,N′,N′-tetramethylguanidinium-polyethylenimine (TP) polymers via a one-step reaction and these polymers were evaluated for efficient delivery of DNA* in vitro* and the results were compared with previously reported 6-(N,N,N′,N′-tetramethylguanidinium chloride)-hexanoyl-polyethylenimine (THP2) polymer. The projected modification enhanced the buffering capacity and charge density, which resulted in the improved transfection efficiency and cell viability. However, on comparison with the previously reported modified THP2/pDNA complex, the transfection efficiency decreased irrespective of cell viability but offered an added advantage of their easy preparation. From these results, it was inferred that TP polymers are much superior to native bPEI and Lipofectamine in terms of transfection efficacy and cell viability and can be used as efficient vectors for nucleic acids in future applications.

## Figures and Tables

**Table 1:** Determination of percent substitution on bPEI by TNBS assay.

S. No.	Sample	Attempted (%)	Observed (%)
1	TP1	10	3.87
2	TP2	30	5.65
3	TP3	50	7.30
4	TP4	70	16.07
5	TP5	100	19.13

**Table 2:** Size and zeta potential measurements of TP polymers and their pDNA complexes at w/w ratio of 3.3 in water and 10% FBS.

S. No.	Samples	Zeta potential (in mV) ± SD			Average particle size (in nm) ± SD	
		Sample (in H_2_O) (+)	DNA complex (in H_2_O) (+)	DNA complex (in 10% FBS)	DNA complex (in H_2_O)	DNA complex (in 10% FBS)
1	TP1	50.8 ± 2.5	43.2 ± 3.4	−7.9 ± 0.4	244.1 ± 21.7	48.3 ± 3.5
2	TP2	42.9 ± 3.4	38.3 ± 2.6	−9.1 ± 1.1	263.5 ± 26.5	65.6 ± 5.2
3	TP3	35.2 ± 2.1	22.8 ± 2.1	−9.9 ± 1.3	341.3 ± 30.4	79.6 ± 8.1
4	TP4	34.4 ± 3.2	20.6 ± 1.7	−12.4 ± 0.8	423.1 ± 35.8	90.9 ± 7.8
5	TP5	31.7 ± 2.8	16.0 ± 1.2	−18.0 ± 1.8	448.2 ± 42.4	130.0 ± 10.5
6	bPEI	40.2 ± 3.6	27.8 ± 2.3	−16.7 ± 2.1	282.2 ± 26.2	42.3 ± 7.5
